# Pickering Emulsions Enhance Oral Bioavailability of Curcumin Nanocrystals: The Effect of Oil Types

**DOI:** 10.3390/pharmaceutics15051341

**Published:** 2023-04-26

**Authors:** Yuze Sheng, Qin Yu, Yanping Huang, Quangang Zhu, Zhongjian Chen, Wei Wu, Tao Yi, Yi Lu

**Affiliations:** 1 Key Laboratory of Smart Drug Delivery of MOE, School of Pharmacy, Fudan University, Shanghai 201203, China; 22111030060@m.fudan.edu.cn (Y.S.); 22111030015@m.fudan.edu.cn (Q.Y.); wuwei@shmu.edu.cn (W.W.); 2 Shanghai Skin Disease Hospital, Tongji University School of Medicine, Shanghai 200433, China; huangyanping7896@163.com (Y.H.); aajian818@163.com (Z.C.); 3 Fudan Zhangjiang Institute, Shanghai 201203, China; 4 Faculty of Health Sciences and Sports, Macao Polytechnic University, Macau, China; yitao@ipm.edu.mo

**Keywords:** curcumin, nanocrystals, Pickering emulsions, oral bioavailability, BCS IV, lipolysis

## Abstract

Nanocrystals (NCs) have the potential to enhance the oral bioavailability of Class IV drugs in the Biopharmaceutical Classification System (BCS) due to the absorption of the intact crystals. The performance is compromised by the dissolution of NCs. Drug NCs have recently been adopted as solid emulsifiers to prepare nanocrystal self-stabilized Pickering emulsions (NCSSPEs). They are advantageous in high drug loading and low side effects due to the specific drug loading mode and the absence of chemical surfactants. More importantly, NCSSPEs may further enhance the oral bioavailability of drug NCs by impeding their dissolution. This is especially true for BCS IV drugs. In this study, curcumin (CUR), a typical BCS IV drug, was adopted to prepare CUR-NCs stabilized Pickering emulsions using either indigestible (isopropyl palmitate, IPP) or digestible (soybean oil, SO) oils, i.e., IPP-PEs and SO-PEs. The optimized formulations were spheric with CUR-NCs adsorbed on the water/oil interface. The CUR concentration in the formulation reached 20 mg/mL, which was far beyond the solubility of CUR in IPP (158.06 ± 3.44 μg/g) or SO (124.19 ± 2.40 μg/g). Moreover, the Pickering emulsions enhanced the oral bioavailability of CUR-NCs, being 172.85% for IPP-PEs and 152.07% for SO-PEs. The digestibility of the oil phase affected the amounts of CUR-NCs that remained intact in lipolysis and, thus, the oral bioavailability. In conclusion, converting NCs into Pickering emulsions provides a novel strategy to enhance the oral bioavailability of CUR and BCS IV drugs.

## 1. Introduction

About 40% of drugs on the market and 70% of new chemical entities under development are poorly water-soluble [1]. Poor solubility casts a shadow on drug development as it leads to low bioavailability and inter- and intra- individual variability [2,3]. Poor permeability exaggerates the dilemma, resulting in extremely poor bioavailability of class IV Biopharmaceutics Classification System (BCS) drugs. Nanocrystals (NCs) have gained great success in the oral delivery of BCS II drugs by enhancing their dissolution. Our previous studies have indicated that NCs did not dissolve instantly following oral administration [4,5,6,7,8]. Then, intact NCs could transport across the intestinal epithelia and accumulate in major organs, such as the liver, lung, and spleen. Consequently, the application of NCs has been extended to oral delivery of BCS IV drugs recently [2,9,10]. For example, 1.25–~7 folds enhancement in oral bioavailability of curcumin (CUR) has been achieved by NCs [11,12,13]. However, dissolution would decrease the number of intact NCs in the gastrointestinal tract and impair the oral bioavailability of BCS IV drugs.

Lipid-based formulations have been a traditional solution to the oral delivery of BC IV drugs [14,15,16]. The underlying mechanism is related to intestinal lipolysis, which converts lipids into colloidal structures, including multilamellar and unilamellar vesicles, mixed micelles, and micelles, together with bile salts, phospholipids, and cholesterol [17,18]. The incorporation of lipolysis products can increase the solubilization capacity of the colloidal structures by up to 50 folds [19]. The colloidal structures are capable of delivering soluble drugs across the mucus layer to the intestinal epithelial cell layer and promote cellular uptake of either drug molecules or intact vehicles [20,21,22,23]. However, LBFs are challenged with low drug loading and potential toxicity from surfactants in formulations.

Pickering emulsions are emulsions with solid particles at the oil–water interface [24,25]. They are superior to traditional emulsions in terms of safety and stability in the absence of surfactants and the anti-coalescence of the solid particle layers [26,27,28]. In addition to inorganic particles, drug NCs have been adopted as emulsifiers to develop the so-called nanocrystal self-stabilized Pickering emulsions (NCSSPEs) [29,30]. Only three components are included in NCSSPEs: water, oil, and drugs. The toxicity concerns arising from heterogeneous particles are dispelled. Drug loading is significantly improved as cargos are not required to be dissolved in the oil phase. NCSSPEs have shown great potential in improving the oral bioavailability of BCS IV drugs, such as silybin (4-fold) and puerarin (2.6-fold) [29,30].

Albeit an LBF, NCSSPEs may undertake different mechanisms in enhancing the oral bioavailability. Cargos in NCSSPEs are mainly adsorbed on the oil surface as NCs instead of being dissolved in the oil. Since the amount of drug NCs tremendously exceeds the solubilizing limit of the oil phase, the secondary colloidal vehicles from lipolysis may only solubilize a small part of the co-formulated drugs despite their excellent solubilization capacity. Most of the drugs during the lipolysis remain in the nanocrystal form. The absorption of detached NCs from NCSSPEs may be the main reason for the enhanced oral bioavailability of BCS IV drugs instead of the traditional mechanism in LBFs. In addition, the formation of NCSSPEs may impede the dissolution of drug NCs since they are adsorbed on the surface of oil droplets. NCSSPEs are thus supposed to further enhance the oral bioavailability of their nanocrystal counterparts.

To evaluate the potential of NCSSPEs in enhancing the oral bioavailability of BCS IV drugs, CUR, a typical class IV drug with various pharmacological activities [31,32], was selected as the model drug. Due to low solubility and permeability, the oral bioavailability of CUR was only 1% or even lower [33,34,35]. An oral dose of 2–4.5 g daily was suggested in clinical trials to treat psoriasis [36,37], which increased the risk of side effects. Different nanoparticles, e.g., micelles, micro/nanoemulsions, nanoparticles, and liposomes, have been developed to enhance the bioavailability of CUR [38]. Incorporation into the nanoparticles improves the solubility of CUR, while the transport of the nanoparticles enhances the permeability of CUR. However, the low loading efficiency limits the application of nanoparticles. Although CUR-NCs are advantageous in high loading, their performance may be compromised by dissolution. By contrast, it was hypothesized that converting CUR-NCs into NCSSPEs may further improve the oral bioavailability of CUR-NCs by impeding the dissolution of the NCs and strengthening epithelial transport. In addition, the digestibility of oils constituting NCSSPEs might have an effect on regulating the release of NCs. Therefore, CUR-NCs stabilized Pickering emulsions were developed in the study with an indigestible (isopropyl palmitate, IPP) and a digestible (soybean Oil, SO) oil, i.e., IPP-PEs and SO-PEs. A pharmacokinetics study was conducted to assess the bioavailability enhancement of the two Pickering emulsions via oral administration. In vitro lipolysis study was performed to explain the different bioavailabilities of the two preparations.

## 2. Materials and Methods

### 2.1. Materials

Curcumin (purity > 97%) was purchased from TCI Shanghai Chemical Industry Co., Ltd. (Shanghai, China), and the internal standard (Emodin, purity > 98%) was purchased from Dalian Meilunbio. Co., Ltd. (Dalian, China). Hydroxypropyl methylcellulose E5 (HPMC E5) was a gift from Shanhe Medical Materials Co., Ltd. (Huainan, China). IPP was obtained from Guangzhou Hefu Chemical Technology Co., Ltd. (Guangzhou, China). SO was obtained from Shandong Ruisheng Pharmaceutical Excipients Co., Ltd. (Liaocheng, China). Sesame oil was obtained from Sigma-Aldrich (Shanghai, China) Trading Co., Ltd. (Shanghai, China). Campul MCM and Captex 355 were gifts from IMCD (China) Co., Ltd. (Shanghai, China). Glyceryl monooleate was obtained from KarmaChem (Shanghai, China). Fasted-state simulated intestinal fluid powder was purchased from Biorelevant (London, UK). Pancreatin was purchased from Shanghai Aladdin Biochemical Technology Co., Ltd. (Shanghai, China), and the activity of amylase and trypsin was 100–350 U/mg, while the lipase activity was 10–75 U/mg. HPLC-grade methanol was purchased from Fisher Chemicals (UK). Other solvents and chemicals were of analytical grade.

Male Sprague Dawley rats (200 ± 20 g) were purchased from JieSiJie Laboratory Animal Co., Ltd. (Shanghai, China). The rats were housed in the animal facility (22–25 °C, 50–60% relative humidity, and 12 h cycle of light and dark) with unlimited access to food and water and fasted for 12 h but free to water prior to the experiment. Our animal experiments were approved by the institutional ethics committee of Shanghai Skin Disease Hospital and were performed in compliance with institutional guidelines (protocol code: 2021-108, date of approval: 29 June 2021).

### 2.2. Preparation of CUR-NCs

CUR-NCs were prepared with minor modifications to the previous method [39]. In brief, 100 mg CUR was dissolved in 5 mL acetone and poured into 100 mL deionized water under magnetic stirring at 500 rpm. The stirring lasted for 10 min, and the acetone was evaporated (R3, Buchi Labortechnik AG, Basel, Switzerland) to obtain coarse CUR dispersions. The dispersions were filtered through a 0.45 µm polycarbonate membrane to remove the water. The coarse CUR crystals trapped by the membrane were resuspended in 50 mL of 0.1% (*w*/*v*) HPMC E5 solution and subsequently processed with a high-pressure homogenizer (Nano DeBEE, BEE International Inc., South Easton, MA, USA) for 15 cycles at 20,000 psi to obtain nanosuspensions. The nanosuspensions were pre-frozen under −80 °C for 4 h and then lyophilized for 48 h to obtain solid CUR-NCs. The powders were stored in hermetic containers before use.

### 2.3. Characterization of CUR-NCs

The size, polydispersity index (PDI), and zeta potential of CUR-NCs were measured by a Malvern Zetasizer Nano (Malvern Instruments, Malvern, UK). CUR-NC suspensions were diluted 10-fold by deionized water before tests. The diluted CUR-NC suspensions were added into folded capillary cells with liquid level over electrodes for zeta potential tests. The morphology of CUR-NCs was observed by scanning electron microscopy (SEM) (SU8010, Hitachi, Tokyo, Japan). Before observation at an accelerating voltage of 1.0 KV, CUR-NC powder was sputter-coated with gold.

### 2.4. Screening of Oils

#### 2.4.1. Solubility of CUR

Five milligrams of CUR were dispersed in 2 g of liquid oils (SO, glyceryl monooleate, Capmul MCM, Captex 355, sesame oil, and IPP). The oil suspensions were placed in a water bath at 37 °C and shaken at 100 rpm for 24 h (HZ-9211KB, Taicang Hualida Laboratory Equipment Co., Ltd., Suzhou, China). After centrifugation for 10 min at 5000× *g*, 0.2 g supernatant was withdrawn and diluted to 10 mL with ethanol. The absorbance of CUR was measured with a UV-Vis spectrophotometer (Lambda 365, PerkinElmer, Waltham, MA, USA) at 425 nm to determine the solubility of CUR. The solubility of CUR in FaSSIF was also determined.

#### 2.4.2. Screening of Oils

Various oils were mixed with CUR-NC suspensions (10 mg/mL) at a ratio of 5:1 and dispersed by high shear at 13,000 rpm for 5 min in a high-speed homogenizer (T18, IKA, Staufen, Germany). The obtained emulsions were diluted 2-fold with deionized water and then dropped onto microscope slides and covered with coverslips, which were eventually observed with optical microscopy (HaikangHuazhi Biotechnology, Suzhou, China) to screen oils with CUR-NC adsorptive capacity on their droplet surface.

### 2.5. Preparation of IPP-PEs and SO-PEs

IPP, an indigestible lipid, and SO, a digestible lipid, were selected as the oil phase to prepare Pickering emulsions. CUR-NCs were dispersed in deionized water as the water phase and then mixed with the oil phase (IPP or SO). The water–oil mixture was then processed with high shear at 13,000 rpm for 5 min in a high-speed homogenizer (T18, IKA, Germany) to obtain CUR-NCSSPEs. Parameters including CUR concentrations (10 mg/mL, 20 mg/mL, 40 mg/mL) and the volume ratios of water: oil phase (1:2, 1:1, 2:1) were screened based on the size of the Pickering emulsions measured by Mastersizer 3000 (Malvern, UK).

### 2.6. Morphology

The morphologies of IPP-PEs and SO-PEs were observed by optical microscopy (HaikangHuazhi Biotechnology, China), confocal laser scanning microscopy (CLSM) (LSM 710, Zeiss, Jena, Germany), and cryo-scanning electron microscopy (Cryo-SEM).

IPP-PEs and SO-PEs were diluted 2-fold with deionized water, which were then dripped onto microscope slides and covered with coverslips. Observed with the optical microscope, images were captured.

Prior to CLSM imaging, Nile Red solution (0.1 mg/mL in alcohol) was used to label the oil phase of IPP-PEs and SO-PEs. Twenty microliters of Nile Red solution were fully mixed with 0.5 mL Pickering emulsions. The stained Pickering emulsions were then diluted 2-fold with deionized water, which were dripped onto microscope slides and covered with coverslips. The slides were then observed under CLSM.The fluorescence of CUR and Nile Red was excited at wavelengths of 488 nm and 561 nm, respectively. The resolution of the scan was set to 1024 × 1024 pixels.

For Cryo-SEM, the method was according to a previous study [40]. Samples were spread onto the perforated stub and freeze-dried (ES-2030, Hitachi, Tokyo, Japan). Then, samples were sputter-coated with gold by an ion sputter (E-1010, Hitachi, Tokyo, Japan) and observed by SEM (SU8010, Hitachi, Tokyo, Japan) under a high vacuum with an acceleration voltage of 5.0 kV.

### 2.7. Pharmacokinetics Study

Male Sprague Dawley rats (200–220 g) were fasted overnight before administration but were allowed free access to water. CUR-NCs (n = 6), IPP-PEs (n = 6), and SO-PEs (n = 4) were intragastrically administered to rats at a dose of 100 mg/kg, respectively. Blood samples were collected at predetermined time intervals (10, 20, 30 min, 1, 2, 4, 6, 8, 10, and 24 h) post-dose and placed into pre-heparinized microcentrifuge tubes immediately. Blood samples were centrifuged at 4000 rpm for 10 min at 4 °C. Plasma was kept frozen at −20 °C pending analysis.

The liquid–liquid extraction method was used to extract CUR from the plasma. Briefly, a 10 μL of emodin solution (2.442 ng/mL), the internal standard, was mixed with a 100 μL plasma sample. Then, a 1 mL mixture of ethyl acetate and methanol containing 0.1% (*v*/*v*) formic acid (9:1, *v*/*v*) was added into the plasma, which was vortex-mixed for 5 min. Following centrifugation at 6000 rpm for 10 min, the organic layer was collected and evaporated under nitrogen flow at 45 °C. Sixty microliters of the mobile phase were added to the residues and vortex-mixed for 30 s to dissolve the extracted CUR. Following centrifugation at 12,000 rpm and 4 °C for 20 min, the supernatant was obtained for LC–MS/MS analysis.

The main pharmacokinetic parameters, such as the area under the concentration–time curve (*AUC*_0–t_), were obtained using compartmental analysis by the DAS 2.0 software package (Mathematical Pharmacology Professional Committee of China, Shanghai, China). The values of peak blood concentration (*C*_max_) and peak time (*T*_max_) were obtained directly from raw data. One-way ANOVA was used to test the differences between groups, and *p* < 0.05 was considered to be a significant difference.

### 2.8. Determination of CUR in Rat Plasma by HPLC-MS/MS

The concentrations of CUR in rat plasma were determined by HPLC-MS/MS (Thermo Scientific, TSQ Quantis and TSQ ALtis, Waltham, MA, USA). CUR was separated by an Agilent Hypersil GOLD C18 (1.9 μm, 100 × 2.1 mm) column at 25 °C. The mobile phase composed of methanol: water (80:20, *v*/*v*) was pumped at a flow rate of 0.2 mL/min. The detection was performed by a Thermoscientific triple quadrupole detector. The mass spectrometer was operated with an electrospray ionization (ESI) interface in negative ionization mode and multiple reaction monitoring modes. The selected reaction monitoring of CUR and emodin was *m*/*z* 366.95/216.97 and *m*/*z* 268.96/224.99, respectively. The peak area ratio of CUR to emodin (*R*) was used for quantification. The linearity was observed within the concentration (*C*) range from 0.5525 to 11.05 ng/mL with a typical calibration equation: *R* = 0.0998*C* − 0.0368, *R*^2^ = 0.9966 (Supplementary Materials Figure S1). Intra-day and inter-day precision (Supplementary Materials Table S1) and accuracy (Supplementary Materials Table S2) all meet the requirements of biological analysis. 

### 2.9. In Vitro Lipolysis Study

The in vitro lipolysis of Pickering emulsions was conducted in the pH-stat lipolysis model with some modifications to the proposed procedure [41].

FaSSIF was prepared by dissolving 0.224 g of biological powder in 100 mL buffer solution (0.126 g of sodium hydroxide pellets, 1.031 g of sodium phosphate monobasic anhydrous, and 1.856 g of sodium chloride dissolved in 300 mL purified water with pH adjusted to 6.5). The FaSSIF stood for 2 h at room temperature before use. Twenty milliliters of FaSSIF were utilized to dissolve 0.2 g of pancreatin, which was then centrifuged at 1000× *g* for 5 min to obtain a supernatant known as the pancreatin solution.

Before lipolysis, two milliliters of pancreatin solution were mixed with 20 mL of FaSSIF. The pH was adjusted to 6.5 with 0.1 M NaOH, which was thermostatically kept at 37 °C in a water bath and stirred at 800 rpm. Then, 1 mL of CUR-NCs, IPP-PEs, and SO-PEs, equivalent to 20 mg/mL CUR, were added to initiate in vitro lipolysis. The pH of the system was maintained at 6.5 with a titration of 0.1 M NaOH. Upon predetermined time intervals (15, 30, and 90 min), five hundred microliters of sample were withdrawn and separated into different phases by centrifuging (centrifuge 5418R, Eppendorf, Hamburg, Germany) at 1000× *g* for 10 min. Each phase was separated and dissolved with ethanol. The content of CUR was measured with the HPLC.

## 3. Results and Discussion

### 3.1. Characterization of CUR-NCs

**Figure 1:**
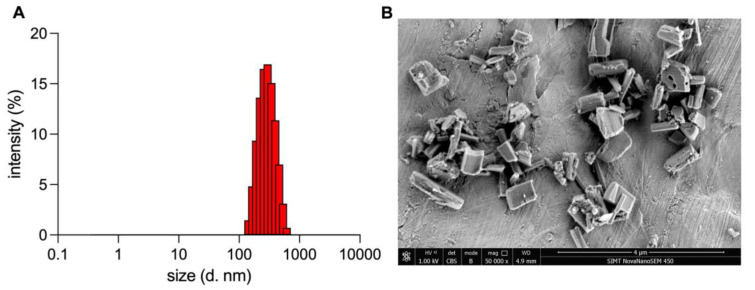
The (**A**) size and (**B**) morphology of CUR-NCs.

The prepared CUR-NCs represented a uniform rod shape, with a mean size of 272.13 ± 2.65 nm, PDI of 0.080 ± 0.001 (Figure 1), and Zeta potential of +23.21 ± 2.10 mV.

### 3.2. Screening of Oil Phase

**Figure 2:**
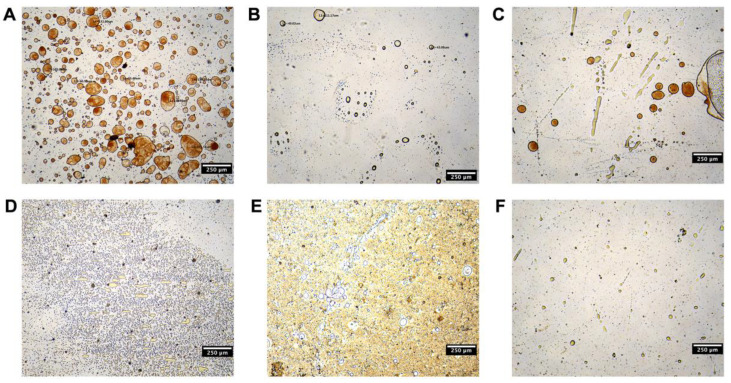
The optical microscopy images of emulsions prepared with (**A**) IPP, (**B**) sesame oil, (**C**) SO, (**D**) Campul MCM, (**E**) glyceryl monooleate, and (**F**) Captex 355.

The solubility values of CUR in oils and Biorelavent fluids are listed in Table 1. CUR showed maximum solubility in Capmul MCM. Nonetheless, the value is within the slightly soluble range, indicating the poor solubility of CUR. CUR showed poorer solubility in sesame oil, IPP, and SO than in other oils. Since CUR-NCs should adsorb on the oil surface to stabilize the Pickering emulsions instead of being dissolved in the oil phase, sesame oil, IPP, and SO are preferable to other oils. Moreover, we evaluated the adsorptive capacity of CUR-NCs on the oil droplets (Figure 2). The wettability of particles is a prerequisite of Pickering emulsion formation and serves a key role in regulating the stability of Pickering emulsions. Particles are required to be partially immersed in both water and oil phases in order to form Pickering emulsions. As the hydrophilic CUR-NCs were dispersed in water, their wettability in different oils contributed to their distinct capacity of being adsorbed on the interface of emulsions. CUR-NCs could only effectively adsorb on IPP and SO to stabilize the oil droplets, while phase separation and inadequate adsorption were observed when mixed with other oils. Thus, indigestible IPP and digestible SO were selected as the oil phase.

### 3.3. Preparation of IPP-PEs and SO-PEs

**Figure 3:**
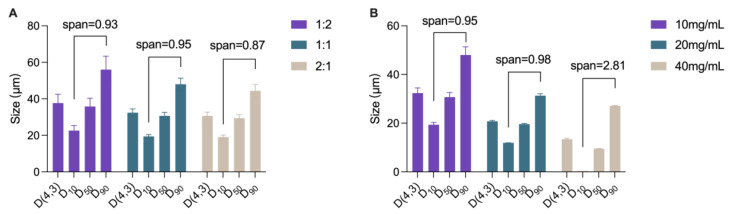
The size variation of IPP-PEs prepared with different (**A**) water: oil ratios and (**B**) CUR concentrations. D(4,3) refers to mass or volume moment mean diameter. D10, D50, and D90 refer to the diameter below which there are 10%, 50% and 90% of particles.

The volume ratios of water to oil phase were set as 1:2, 1:1, and 2:1 to investigate their effects on the size and size distribution of IPP-PEs. The results showed that the sizes of IPP-PEs tended to decrease with the increasing proportion of water. When the ratio was at 1:1 and 2:1, D(4,3), D10, D50, and D90 were comparable and lower than those of 1:2 with lower SD values (Figure 3A). Considering the benefit of oils in terms of solubilization and absorption promotion, the ratio of 1:1 was selected. Then, the drug concentration was screened at the level of 10 mg/mL, 20 mg/mL, and 40 mg/mL with the volume ratio of water to oil phase set as 1:1 (Figure 3B). The size decreased with the increase in CUR concentration from 10 mg/mL to 20 mg/mL due to the increased amount of solid particle emulsifiers. However, the size was nonuniformly distributed when the CUR concentration was further increased to 40 mg/mL. The reason may be attributed to the agglomeration among NCs and/or Pickering emulsions. In contrast, 20 mg/mL was better for the narrower size distribution and smaller droplet size. Thus the CUR concentration of 20 mg/mL, along with the water: oil phase ratio of 1:1, was selected as the optimal formulation of IPP-PEs. SO-PEs were prepared in the same process as IPP-Pes, yet the oil phase was substituted with an equal amount of SO. Of note is that the CUR concentration in the preparation far outweighed its solubility in the oil phase (Table 1).

### 3.4. Characterization of IPP-PEs and SO-PEs 

#### 3.4.1. Size distribution

**Figure 4:**
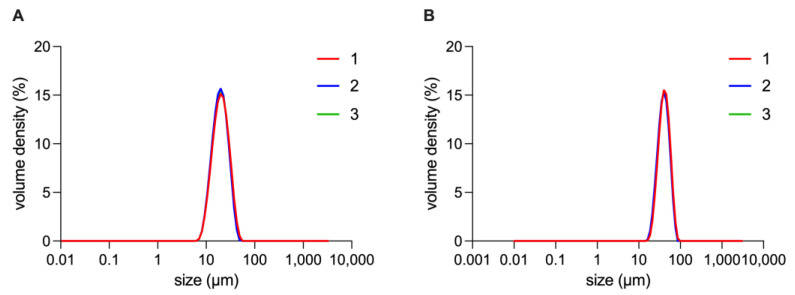
The size distribution of (**A**) IPP-PEs and (**B**) SO-PEs. 1, 2, and 3 represents the triplicate measurement.

The size distribution of IPP-PEs and SO-PEs is shown in Figure 4. Their mean size was 20.77 ± 0.40 μm and 44.3 ± 0.85 μm, and their D10, D50, D90 was 27.83 ± 0.29 μm, 42.37 ± 0.40 μm, 62.93 ± 0.46 μm and 11.90 ± 0.08 μm, 19.60 ± 0.28 μm, 31.3 ± 0.82 μm, respectively.

#### 3.4.2. Microstructures

**Figure 5:**
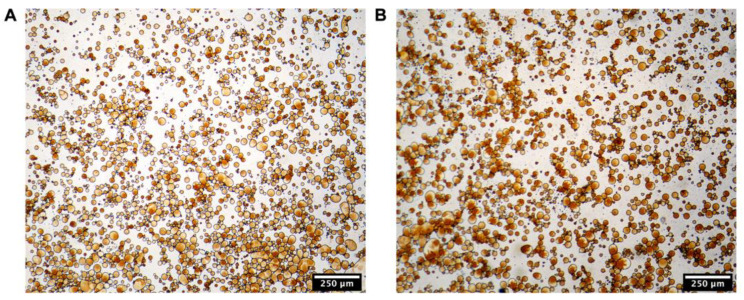
The optical microscopy images of (**A**) IPP-PEs and (**B**) SO-PEs.

The optical micrographs of IPP-PEs and SO-PEs are displayed in Figure 5. Most of them present spherical morphology, and their diameters were consistent with the results of Section 3.4.1.

**Figure 6:**
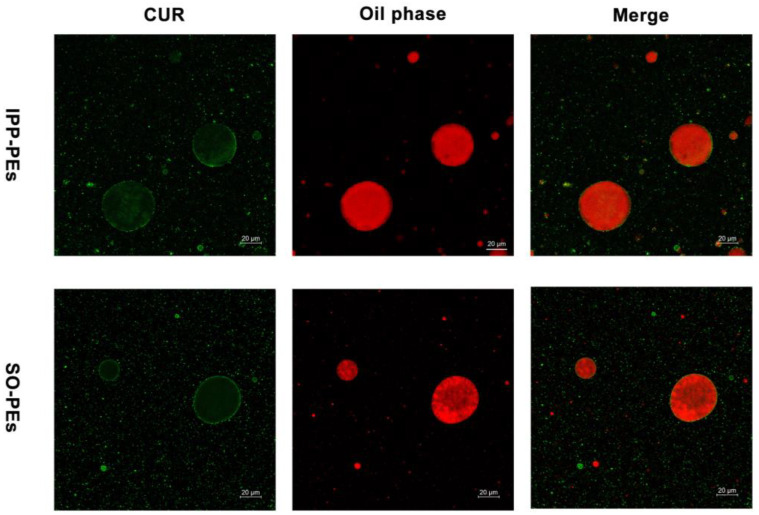
The CLSM images of IPP-PEs and SO-PEs. Green represents CUR and Red represents Nile Red stained IPP and SO.

To confirm that CUR-NCs adsorb on the water/oil interface, the oil phase of the Pickering emulsions was stained using Nile Red for imaging with CLSM (Figure 6). Nile Red is an extremely lipophilic dye that has been widely used to stain the oil phase of Pickering emulsions [42], emitting red fluorescence (Figure 6). CUR itself is a fluorescent substance and emits green fluorescence. Based on the green signal observed from CLSM, CUR-NCs mainly adsorbed on the water/oil interface of the Pickering emulsions. Faint green signals were seen in the oil core, which was due to the dissolved CUR. Despite the interference from the strong signal of Nile Red, the green shell of CUR-NCs could be identified in the merge channel of CLSM, supporting the adsorption of CUR-NCs on the surface of oil droplets. 

**Figure 7:**
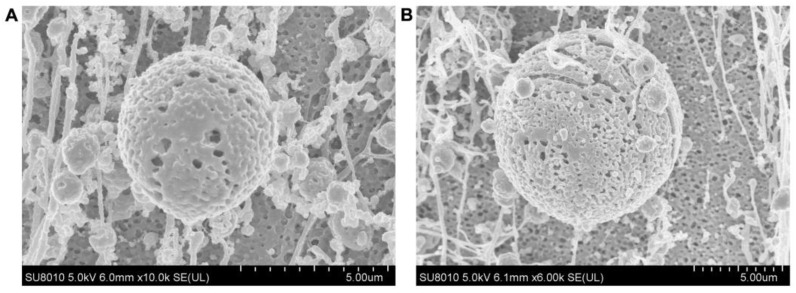
The Cryo-SEM images of (**A**) IPP-PEs and (**B**) SO-PEs.

In addition, the microstructures of IPP-PEs and SO-PEs were observed by Cryo-SEM (Figure 7). Both IPP-PEs and SO-PEs were spherical. The surface of the oil droplet was full of bumps and holes, indicating the adsorption of CUR-NCs.

### 3.5. Pharmacokinetics Study

**Figure 8:**
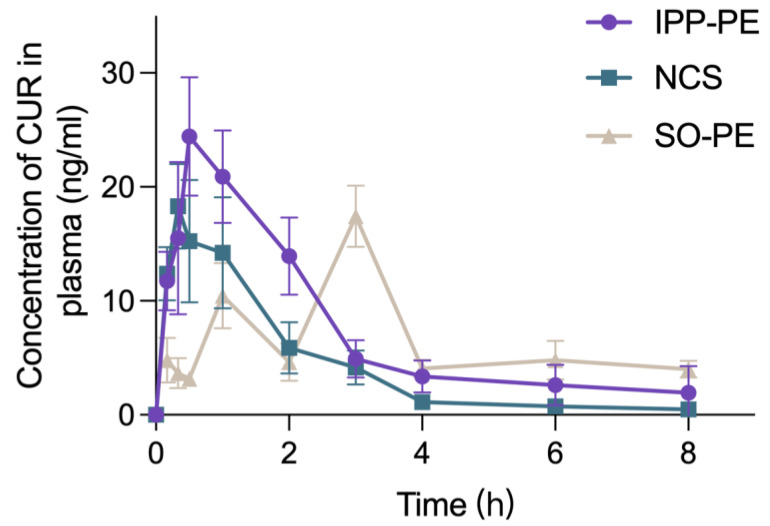
The Plasma concentration–time curves of IPP-PEs (n = 6), SO-PEs (n = 4), and CUR-NCs (n = 6) after a single administration at the CUR dose of 100 mg/kg in rats. (mean ± SD).

The plasma concentration–time curves of IPP-PEs, SO-PEs, and CUR-NCs are presented in Figure 8, while the main pharmacokinetic parameters are listed in Table 2. In addition to the poor solubility and permeability, CUR suffers extensive first-pass metabolism. Albeit a high dose of 100 mg/kg, the plasma concentration of CUR fell below the limit of quantitation at 10 h post-administration. CUR-NCs achieved the shortest *T*_max_ and moderate *C*_max_ among all preparations. Since CUR belongs to BCS IV drugs, the absorption may be attributed to transepithelial transport of the intact NCs instead of diffusion of the dissolved molecules. However, dissolution led to the reduction of intact NCs. After a quick absorption and reaching a moderate peak concentration, the plasma concentration of CUR decreased, producing the lowest *AUC*_0–8_ among all preparations. IPP-PEs achieved a similar pharmacokinetic profile to CUR-NCs except for a longer *T*_max_ of 0.75 ± 0.25 h and a higher *C*_max_ of 26.78 ± 3.54 μg/L. The curves in the absorption phase from the two preparations are almost superposed. It is inferred that transepithelial transport of detached CUR-NCs may be the main reason for the absorption of IPP-PEs. Since IPP is indigestible, the structure of Pickering emulsions may hinder the dissolution of CUR-NCs due to the interaction with the oil. The continuous transport led to the higher *C*_max_ and the lag of *T*_max_. Consequently, the oral bioavailability of IPP-PEs reached 172.85% relative to CUR-NCs. SO-PEs achieved a different pharmacokinetic profile, presenting triple peaks. The first peak at 10 min may be due to the fast absorption of unadsorbed CUR-NCs in the preparation. CUR-NCs may have stronger interaction with SO than IPP. However, SO is digestible. CUR-NCs may be gradually detached from SO-PEs with the lipolysis of SO. The subsequent absorption of detached NCs led to multiple peaks. Therefore, SO-Pes achieved the longest *T*_max_ among the preparations. Although SO-PEs achieved a lower *C*_max_ than CUR-NCs, the *AUC* and oral bioavailability were enhanced due to the extended absorption period. Nonetheless, IPP-PEs had a higher oral bioavailability than SO-PEs, being 172.85% versus 152.07%. Of note is that the oral bioavailability is relative to NCs. An even higher bioavailability can be anticipated in comparison with CUR coarse crystals. The pharmacokinetics study proved the efficiency of Pickering emulsions in promoting oral absorption of CUR-NCs, which may apply equally to other BCS IV drugs.

### 3.6. In Vitro Lipolysis Study

**Figure 9:**
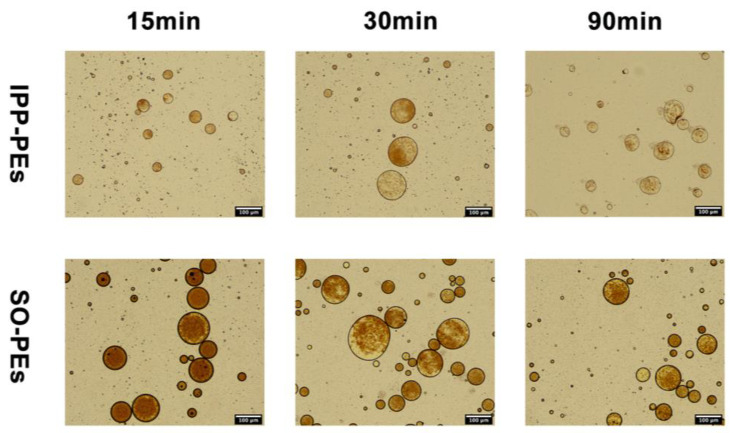
The optical microscopy images of morphologic transformation of IPP-PEs and SO-PEs during lipolysis.

The pH-stat lipolysis model was adopted to estimate the intestinal digestion of Pickering emulsions. The morphology transformation of IPP-PEs and SO-PEs during the lipolysis was shown in Figure 9. The dynamic process of CUR-NCs detached from the surface of Pickering emulsions was evidently displayed, IPP-PEs exhibiting a faster peeling rate compared with SO-PEs, which may explain the difference between the plasma concentration-time curves of IPP-PEs and SO-PEs. The slower CUR-NCs detaching rate of SO-PEs resulted in the lower plasma concentration increase before the second peak and with the SO digestion completed large amounts of CUR-NCs were released contributing to the third peak.

**Figure 10:**
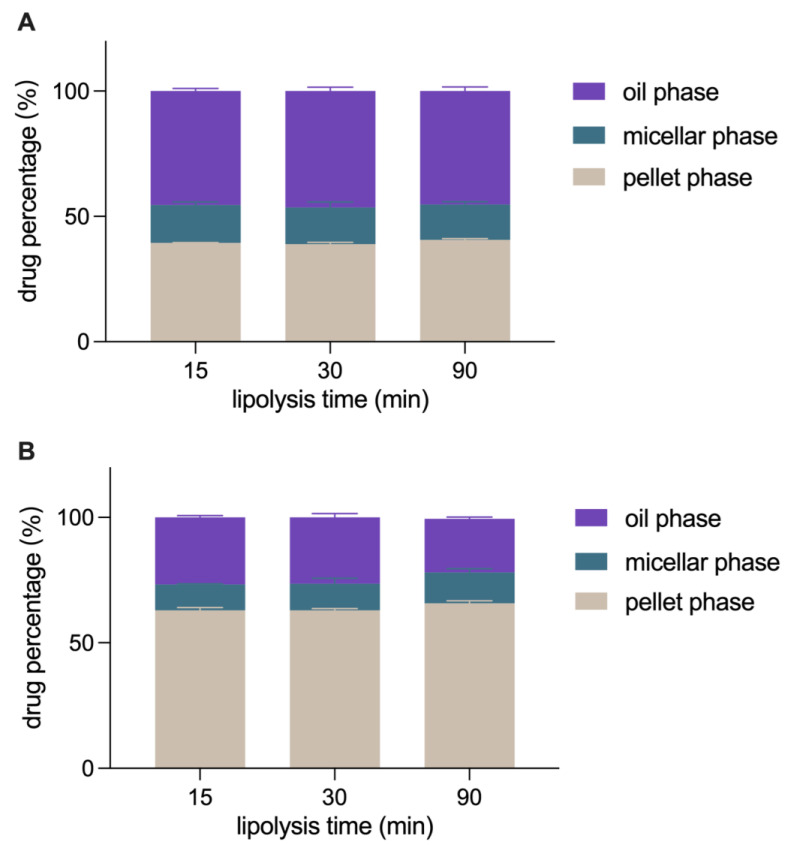
The in vitro lipolysis profiles of (**A**) IPP-PEs and (**B**) SO-PEs in FaSSIF.

Figure 10 show the distribution of CUR in the lipolysis solution of IPP-PEs and SO-PEs. Three phases are obtained by centrifugation of the lipolysis solution, i.e., the bottom pellet phase containing precipitates, the middle micellar phase containing solubilized CUR, and the top oil phase containing undigested oil and CUR-NCs. During the in vitro lipolysis of CUR-NCs, 93.92% of CUR precipitated, while only 6.08% was dissolved in the micellar phase (data not shown in Figure 10). The reason was attributed to the poor solubility of CUR in FaSSIF, only 2.92 μg/mL. The quick dissolution of CUR-NCs produced a supersaturated solution. After the crystallization induction time, CUR precipitated from the solution and induced the growth of undissolved CUR-NCs. Finally, the big crystals were collected in the pellet phase following centrifugation. Conversely, the percentage of CUR in the pellet phase was 39–41% and 62–66% in the lipolysis solution of IPP-PEs and SO-PEs, respectively. It is inferred that IPP and SO could retard the dissolution of CUR-NCs from being detached during lipid digestion. In addition, the percentage of CUR content in the micellar phase was 14.07% and 12.77% in the lipolysis solution of IPP-PEs and SO-PEs, respectively. They were much higher than that of CUR-NCs (6.08%). The reason is due to the incorporation of lipolysis products of oil that increases the solubilization capacity of the micellar phase. IPP is similar to 2-monoglyceride in structure, which is the main component in the lipolysis of triglyceride products with enhanced surface activity. In this regard, IPP may also be incorporated into the micellar phase to enhance the solubilizing capacity. Moreover, CUR content in the oil phases of the lipolysis solution of IPP-PEs almost kept invariant, being ~46%, because IPP could not be broken down by pancreatic lipase. On the contrary, with the lipolysis of SO, the CUR content in the oil phase decreased from 26.79% to 21.44%, while CUR content in the micellar phase and pellet phase accordingly increased. Since CUR-NCs retained in the oil phase were significantly higher than CUR dissolved in the micellar phase, absorption of CUR-NCs is the main reason for the enhanced oral bioavailability of the Pickering emulsions. IPP is stronger than SO in maintaining CUR-NCs in the oil phase and solubilizing CUR in the micellar phase. Consequently, IPP-PEs achieved a higher bioavailability than SO-PEs.

## 4. Conclusions

CUR-NCs could be prepared via a homogenization method. Based on the solubility and interaction, IPP and SO were selected to prepare CUR-NC-stabilized Pickering emulsions, respectively. IPP-PEs and SO-Es were spheric, while CUR-NCs were adsorbed on the water/oil interface. Therefore, CUR concentration in the formulation was far beyond CUR solubility in IPP and SO. The Pickering emulsions enhanced the oral bioavailability of CUR-NCs by 1.73- (IPP-PEs) and 1.52- (SO-PEs) fold, respectively. The difference may be due to the digestibility of the oil phase. The indigestible IPP maintained a higher percentage of intact CUR-NCs than the digestible SO in in vitro lipolysis, leading to a higher AUC and a shorter *T*_max_. To sum up, the NCSSPEs delayed the dissolution of CUR-NCs and provided more opportunities for the intact NCs to be absorbed, standing for a novel strategy to enhance the oral bioavailability of CUR and BCS IV drugs.

## Figures and Tables

**Table 1:** The solubility of CUR in different oils and in FaSSIF (n = 3, mean ± SD).

Solvent	Solubility
SO	124.19 ± 2.40 (μg/g)
glyceryl monooleate	217.66 ± 5.34 (μg/g)
Capmul MCM	1323.03 ± 25.87 (μg/g)
Captex 355	557.78 ± 10.23 (μg/g)
sesame oil	42.49 ± 1.02 (μg/g)
IPP	158.06 ± 3.44 (μg/g)
FaSSIF	2.92 ± 0.02 (μg/mL)

**Table 2:** Pharmacokinetic parameters of IPP-PEs (n = 6), SO-PEs (n = 4), and CUR-NCs (n = 6) after a single administration at the CUR dose of 100 mg/kg in rats. (mean ± SD).

	CUR-NCs	SO-PEs	IPP-PEs
*T*_max_ (h)	0.50 ± 0.24	3.00 ± 0.00	0.75 ± 0.25
*C*_max_ (μg/L)	21.18 ± 2.82	17.43 ± 2.69	26.78 ± 3.54 *
*AUC*_0–8_ (μg/L*h)	34.22 ± 7.56	52.04 ± 7.58 *	59.15 ± 8.36 ***
F_rel_ (%)	/	152.07	172.85

* *p* < 0.05, *** *p* < 0.001 vs. CUR-NCs group.

## Data Availability

Data available upon request due to restrictions, e.g., privacy or ethical.

## Supplementary Materials

The following supporting information can be downloaded at: https://www.mdpi.com/article/10.3390/pharmaceutics15051341/s1. Figure S1. The standard curve for detection of CUR in plasma. Table S1. The intra-day and inter-day precision for detection of CUR in plasma. Table S2. The accuracy for detection of CUR in plasma.
