# Supplementary material for: Pickering Emulsions Enhance Oral Bioavailability of Curcumin Nanocrystals: The Effect of Oil Types
Source: Pharmaceutics. 2023 Apr 26;15(5):1341. doi: 10.3390/pharmaceutics15051341 (PMC10221895; doi:10.3390/pharmaceutics15051341)
Supplement: Supplementary file 1 [file pharmaceutics-15-01341-s001.zip › pharmaceutics-2316422-supplementary.pdf]

Article

Pickering emulsions enhance oral bioavailability of curcumin nanocrystals: The effect of oil types

Supplementary Materials

Yuze Sheng [1,†], Qin Yu [1,2,†], Yanping Huang [2], Quangang Zhu [2], Zhongjian Chen [2], Wei Wu [1,2,3], Tao Yi [4] and Yi Lu [1,2,3,*]

[1] Key Laboratory of Smart Drug Delivery of MOE, School of Pharmacy, Fudan University, Shanghai 201203, China
[2] Shanghai Skin Disease Hospital, Tongji University School of Medicine, Shanghai 200433, China
[3] Fudan Zhangjiang Institute, Shanghai 201203, China
[4] Faculty of Health Sciences and Sports, Macao Polytechnic University, Macau, China
* Correspondence: fd_luyi@fudan.edu.cn
† The authors contributed equally to this work.

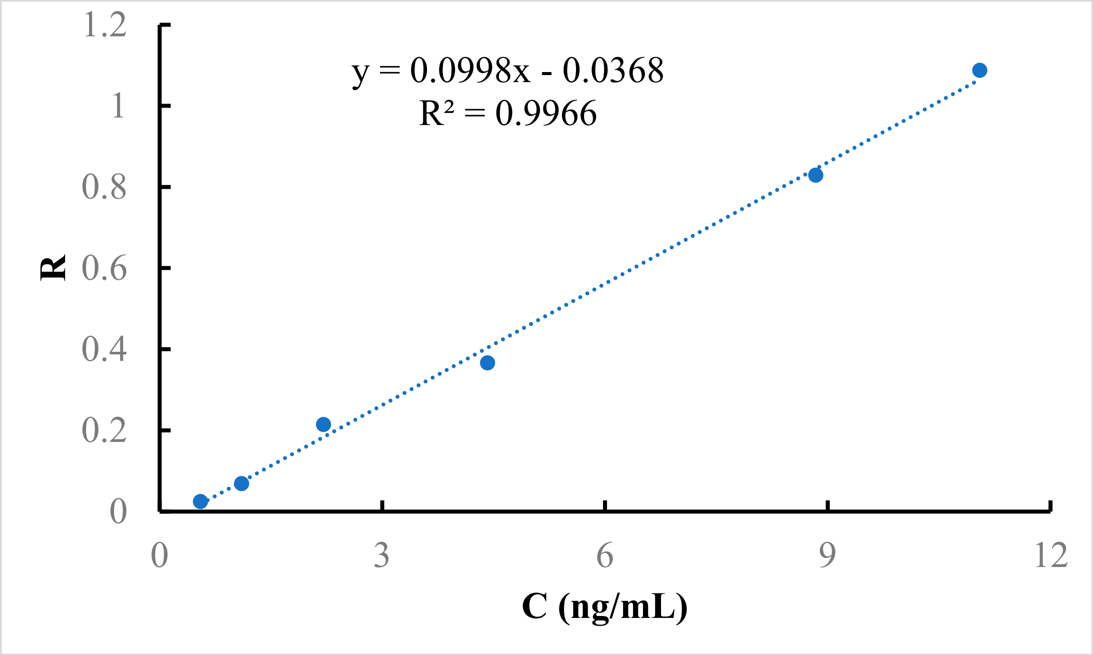

Figure S1. The standard curve for detection of CUR in plasma.

Table S1. The intra-day and inter-day precision for detection of CUR in plasma.

Concentration (ng/mL)	Intra-day RSD (%)	Inter-day RSD (%)
1.105	9.089	9.471
4.420	4.190	5.533
8.840	7.569	8.414

Table S2. The accuracy for detection of CUR in plasma.

Concentration (ng/mL)	Detected concentration (ng/mL)	Accuracy (%)
1.105	1.757 ± 0.099	97.346 ± 8.848
4.420	4.005 ± 0.168	90.611 ± 3.797
8.840	7.981 ± 0.603	90.288 ± 6.826
